# An attention-based transfer learning framework for breast cancer classification in mammography under limited data conditions

**DOI:** 10.3389/fmedt.2026.1842859

**Published:** 2026-07-09

**Authors:** Niketa Banyal, Aman Kumar

**Affiliations:** Department of Electronics and Communication Engineering, National Institute of Technology Hamirpur, Hamirpur, Himachal Pradesh, India

**Keywords:** attention mechanism, breast cancer, classification, mammography, Squeeze-and-Excitation

## Abstract

Breast cancer is a leading cause of death for women worldwide. There is a critical need for an early and accurate diagnosis to improve survival rates. This study evaluates the performance of transfer learning-based MobileNetV2 and ResNet50 architectures for binary breast cancer classification using mammography images. To enhance channel-wise feature learning, the Squeeze-and-Excitation (SE) attention mechanism was integrated into the network and partial fine tuning was performed by only freezing the early layers of the model. Furthermore, gradient-weighted class activation mapping was applied to visualize the important regions of mammogram images responsible for the model’s predictions. The performance evaluation was carried out on the Contrast Limited Adaptive Histogram Equalization (CLAHE) binary, Digital Database for Screening Mammography (DDSM), INbreast, and Mammographic Image Analysis Society (MIAS) datasets using stratified k-fold cross-validation. The experimental results demonstrate that one of the proposed frameworks, MobileNetV2-SE, achieved mean classification accuracies of 99.14%, 93.53%, 92.35%, and 96.33% on the DDSM, INbreast, MIAS, and CLAHE-binary datasets, respectively. In comparison, the ResNet50-SE model attained mean accuracies of 100%, 88.89%, 71.60%, and 97.98% on the same datasets. The experimental results demonstrate that the frameworks achieve reliable and competitive performance for breast cancer detection under realistic evaluation conditions and improve the diagnostic accuracy of mammogram classification. This work contributes to the field of medical image analysis and computer-aided diagnosis. Future work should focus on applying advanced class balancing methods to improve diagnostic accuracy and model robustness in a variety of medical imaging applications.

## Graphical Abstract

**Figure:**
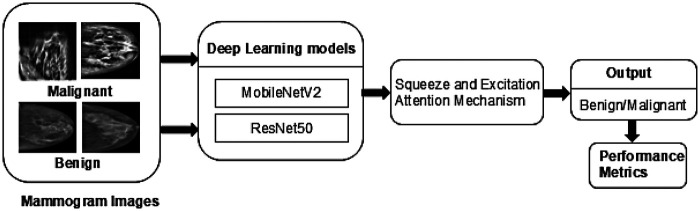

## Highlights

- The MobileNetV2 and ResNet50 models are used as the baseline architectures.
- The Squeeze-and-Excitation attention mechanism is integrated to improve channel-wise feature representation.
- Gradient-weighted class activation mapping-based explainable AI is incorporated to enhance model interpretability and clinical transparency.
- The performance analysis was performed on the individual Digital Database for Screening Mammography (DDSM), INbreast, Mammographic Image Analysis Society (MIAS) datasets, and the Contrast Limited Adaptive Histogram Equalization (CLAHE) binary combined dataset.
- The MobileNetV2 and ResNet50 models achieved higher accuracy up to 99% in all datasets while maintaining computational efficiency.

## 1 Introduction

Breast cancer is the most frequently diagnosed cancer among women globally. According to the WHO in 2022, there were 2.3 million new cases and 670,000 deaths (1). Early detection of breast cancer is very important to improve survival rates (2). There are many imaging modalities for diagnosing breast cancer, such as ultrasound, mammography, MRI, and histopathology, of which mammography is the most popular. The mammogram test helps classify abnormalities as benign or malignant, but the interpretation of mammograms remains a challenge for experienced radiologists. Subtle lesions are often difficult to detect due to the overlapping of tissues and variability in breast density. These factors can lead to missed diagnoses and unnecessary recalls (3). In many regions, the number of screening examinations has increased, and combined with the shortage of experienced radiologists, has further increased the workload of radiologists, the risk of fatigue, and variability in interpretation. To address these challenges, a computer-aided diagnosis (CAD) system has been introduced to assist the radiologist by providing decision support, offering a second opinion, and helping highlight cases that need more examination (4, 5).

In this context, machine learning and deep learning methods are often used to examine medical data and sort it into groups. Conventional breast cancer classification methods commonly employ machine learning algorithms, such as support vector machine (SVM), k-nearest neighbor (KNN), decision trees, and random forest. These methods are computationally efficient, easy to interpret, and require lower computational resources, making them suitable for small-scale applications. However, conventional methods often rely on handcrafted features, have limited ability to capture complex patterns in medical images, and generally show lower accuracy compared to deep learning approaches. These models depend on the features that are manually extracted and work well with structured data.

Deep learning techniques, in contrast, offer more advanced methods because they learn features from raw data. Convolutional neural networks (CNNs) are useful for analyzing medical images because they find spatial and hierarchical patterns. CNNs include convolutional layers, pooling layers, and fully connected layers. These layers work together to find features and perform classification tasks.

The major difference between deep learning and standard machine learning is that deep learning can work with large, complicated datasets without the need for explicit feature engineering. This makes deep learning highly effective when analyzing high-resolution medical images. Despite their advantages, both techniques face certain challenges, such as overfitting, high computational requirements, and the need for large, labeled datasets. Therefore, selecting an appropriate model and optimizing its parameters are crucial steps in creating a reliable breast cancer detection system.

In mammography, CNNs are widely used for both lesion classification and whole image assessment. CNN architectures such as ResNet50 and light models such as MobileNetV2 have gained widespread adoption due to their strong feature extraction capabilities and the availability of pretrained weights on large-scale datasets such as ImageNet. These models have demonstrated strong results when combined with transfer learning (6). By leveraging previously learned visual representations, transfer learning reduces training time and improves convergence while mitigating overfitting to a certain extent. However, despite these advantages, the effective use of deep learning in medical imaging continues to be limited by real-world challenges.

Our major challenge is the limited size and variability of publicly available breast cancer datasets. The medical datasets, i.e., Digital Database for Screening Mammography (DDSM), INbreast, and Mammographic Image Analysis Society (MIAS), contain a limited number of sample images and different resolutions, and often exhibit a class imbalance between benign and malignant cases. This scarcity of datasets restricts the ability of a model to effectively learn discriminative features, especially in the case of complex architectures and attention-based modules. As a result, a model may achieve high performance on training or validation data while struggling to generalize on unseen samples.

To address these challenges, this study evaluates the performance of two pretrained CNN architectures, MobileNetV2 and ResNet50, which are employed as backbone models, with selective fine-tuning applied to balance feature reuse. In addition, a Squeeze-and-Excitation (SE)-based attention mechanism is integrated into the frameworks without introducing excessive computational complexity. The models are trained under a consistent pipeline using the dataset images, with consideration given to class distribution, dataset splits, and evaluation metrics.

To enhance the interpretability of the models, an artificial intelligence (AI) technique, gradient-weighted class activation mapping (Grad-CAM), is incorporated, which highlights the important regions of the mammogram images and helps one understand the decision-making process of the models. The performance of the models was assessed using accuracy, precision, recall, F1 score, and area under the curve (AUC) to provide a comprehensive understanding of classification behavior. This study places a strong emphasis on analyzing model generalization, particularly in limited data availability and diverse dataset distribution scenarios. The primary contribution of this article lies in the robustness and generalization behavior of transfer learning-based CNN models with an integrated SE attention mechanism and Grad-CAM explainability for efficient breast cancer classification using mammogram images from multiple datasets.

## 2 Literature review

This section discusses previous studies that focus on the robustness and consistency of CNN features under rotational transformation. Deep learning-based breast cancer detection in medical imaging has seen significant advances in tumor classification and localization. Several studies have highlighted various methodologies for the detection of breast cancer.

Shen et al. (7) trained a model on a large-scale mammography dataset to improve detection sensitivity and showed improved performance compared to the conventional CAD system. The proposed all convolutional network achieved a per-image AUC of 0.91 on the CBIS-DDSM dataset with 86.1% sensitivity and 80.1% specificity, while on the INbreast Dataset, it achieved an AUC of 0.98 with 86.7% sensitivity and 96.1% specificity after model averaging and fine-tuning. Although performance was good in the detection of breast cancer, the model suffered from limited data diversity and interpretability. Thus, a lack of large-scale clinical validation remains a key gap in the transition of these models to a real-world environment. Sumaiyya et al. (8) utilized a MobileNetV2 CNN model with integrated explainable techniques, achieving an accuracy level between 85% and 87%. The key challenge in this model was also limited dataset diversity and the absence of external validation.

Lanjewar et al. (9) proposed a hybrid framework combining MobileNetV2, ResNet50, and VGG16 with long short-term memory (LSTM) for feature extraction from ultrasound images. To address the class imbalance, the synthetic minority oversampling technique (SMOTE) was applied to the extracted features. The proposed VGG16-based model achieved a high F1 score of 99.0%, Matthews correlation coefficient (MCC) and Kappa score of 98.9%, and an AUC of 1.0. In addition, the model was validated using K-fold cross-validation, achieving an average F1 score of 96%. For model interpretability, the Grad-CAM and local interpretable model-agnostic explanations (LIME) techniques were applied. The key limitations observed in this study were overfitting and the model was only reliable on a single imaging modality, namely, ultrasound. Furthermore, the model exhibited limited explainability, which restricts its real-world applicability. Alshamrani and Alshomrani (6) introduced a dual-framework ResNet50 combined with SMOTE to handle class imbalances in mammogram images. The model achieved 99% accuracy on a balanced dataset and 90% on an imbalanced dataset. However, the model was more complex due to the action of a deep backbone. Nair et al. (10) proposed a SE conformer-based framework for cancer detection in histopathology images. To enhance feature representation, the model combined convolutional and transformer mechanisms. The results indicated improved classification accuracy, precision, and recall of 0.97 compared to conventional CNN models. The primary limitations were high computational demand and the limited testing dataset.

Surya et al. (11) introduced a MobileNetV2-based transfer learning framework to classify breast ultrasound images into normal, benign, and malignant categories. The model achieved 82% accuracy with a receiver operating characteristic (ROC)-AUC of 0.94, offering real-time deployment using Streamlit and demonstrating the feasibility of lightweight CNN architectures for practical applications in oncology diagnostics. Despite its good accuracy, the performance of the model was constrained by a limited dataset size and class imbalances. Taifi et al. (12) improved MobileNetV2, DenseNet-121, and DenseNet-201 models with modified activation functions for breast cancer diagnosis. The enhanced DenseNet-201 model achieved the highest accuracy of 99.6% on the DDSM dataset. Similarly, the modified DenseNet-121 model attained 97% accuracy on MIAS and 98% on INbreast, while the improved DenseNet-201 reached 98% on both MIAS and INbreast. Furthermore, the lightweight yet optimized MobileNetV2 achieved impressive results, with 99.4% accuracy on DDSM and 99% on INbreast. However, despite its higher accuracy, the model introduced more complexity through the introduced Gaussian error linear unit (GELU) activation function, which may affect inference speed in resource-constrained clinical environments.

Rakha et al. (13) proposed a hybrid model, combining MobileNetV2 with a convolutional block attention module (CBAM) to improve cancer detection in ultrasound images, achieving a test accuracy of 93%. The attention mechanisms improved feature extraction. Furthermore, the model had improved tumor classification performance compared to a baseline MobileNetV2 model. However, the computational overhead increased as a result of the additional attention module.

Nissar et al. (14) introduced MOB-CBAM, a dual-channel attention-based deep learning model to predict breast cancer molecular subtypes from mammograms. The study utilized CBAM attention modules to improve discriminative feature learning, but the model’s computational complexity was high because of the dual-channel CBAM attention mechanism. Maurya et al. (15) proposed FCCS-Net, a multi-level channel and spatial attention-based transfer learning framework for breast cancer classification. The model had improved feature extraction and achieved high classification performance using an attention mechanism. Despite notable advancements in breast cancer detection across various datasets, several significant gaps remain. One key challenge is real-time validation. Panambur et al. (16) proposed an attention erasing strategy to enhance transfer learning performance in breast abnormality classification. The method improved the model’s robustness by forcing the network to focus on multiple informative regions in mammograms. This framework strongly depends on the accuracy of attention maps, as incorrect localization may remove important lesion features and reduce classification performance.

Behar and Shrivastava (17) proposed a ResNet50 model for the classification of histopathological images, introducing a transfer learning approach that improved classification accuracy. However, the model was unable to address the imbalanced dataset. Islam et al. (18) proposed a CBAM-optimized ResNet50 model for breast lesion classification on mammograms, achieving an AUC of 0.866 ± 0.015, which significantly outperformed the standard ResNet50 model with an AUC of 0.772 ± 0.008. This model also depended on attention map accuracy, with improper attention mapping leading to the removal of important lesion features. Heikal et al. (19) improved the model’s classification accuracy by fine-tuning the deep learning architecture of breast cancer detection. However, the method was strongly dependent on fine-tuning tailored to a specific dataset, which restricts generalization. Wang et al. (20) also applied the fine-tuning method to a MobileNet model for the classification of breast cancer detection in an MRI dataset. The study highlighted that transfer learning significantly improves diagnostic accuracy. The model was lightweight, but it only works for MRI scans.

To address the black box nature and limited interpretability of deep learning frameworks, explainable AI (XAI) techniques such as Grad-CAM have been widely adopted to provide a visual representation of model prediction (21). Mobini et al. (22) conducted a comparative study on deep transfer learning approaches for detecting breast arterial calcification on mammograms. The study demonstrated the effectiveness of pretrained CNN models and Grad-CAM visualization for medical image interpretation.

Another study by Chakravarthy et al. (23) integrated spatial attention, EfficientNet architecture, and explainable AI techniques for breast cancer classification, achieving a maximum classification accuracy of 97.585% on CBIS-DDSM, 98.255% on INbreast, and 98.91% MIAS on using the proposed ESA-XGBNet architecture. The framework had improved interpretability and enhanced model transparency due to the XAI visualization methods.

Recent studies have also explored deep learning approaches for medical image classification beyond breast cancer applications. A study on nail disease classification proposed a modified DenseNet169 architecture integrated with leaky rectified linear unit (ReLU) activation and LSTM layers for effective feature extraction. The proposed method achieved an F1 score of 89.9%, AUC of 98.2%, MCC of 84.7%, and Kappa score of 84.6% using fivefold cross-validation along with statistical confidence intervals and significance testing (*p* = 0.016), demonstrating strong generalization capability in clinical diagnosis tasks (24). Another study on nail disease classification utilized a modified DenseNet169 architecture combined with LSTM and data balancing techniques, achieving an accuracy of 84.7%, an F1 score of 89.9%, an AUC of 98.2%, and a Kappa score of 84.6%, validated using fivefold cross-validation and statistical confidence interval analysis (*p* = 0.016) (24). These results indicate that the integration of attention mechanisms and hybrid deep learning models significantly improve feature representation, classification accuracy, and generalization performance in medical imaging applications.

Despite strong performance in different medical imaging applications, limited dataset diversity, computational complexity, and clinical reliability are common challenges. Therefore, there is still a need for efficient and optimized attention-based transfer learning frameworks specifically designed for breast cancer classification. The proposed models attempt to address these shortcomings by balancing complexity with usability, improving generalization across datasets, improving transparency, and ensuring clinical relevance.

## 3 Methodology

**Figure 1:**
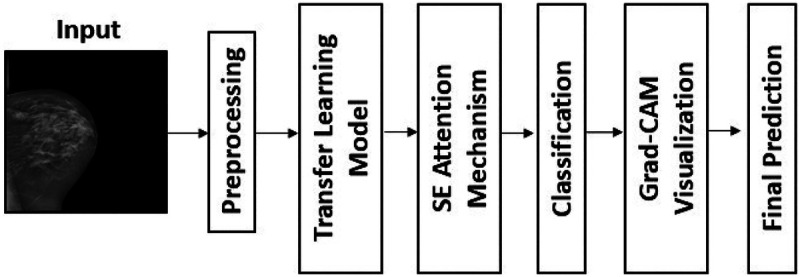
Overall workflow of the proposed breast cancer classification system.

This section presents a comprehensive overview of the mammography image datasets employed in this study and details the deep learning architectures utilized for automated breast cancer detection. The overall workflow is shown in Figure 1.

### 3.1 Dataset selection and preparation

**Figure 2:**
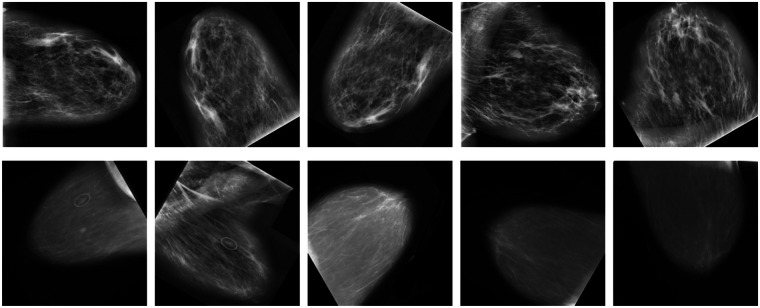
Selected sample images from the CLAHE-binary mammogram dataset illustrating benign and malignant lesions.

Mammography datasets are the standard imaging modality for large-scale screening programs and early identification of breast lesions. These datasets provide images with different resolutions and enable the design of robust and generalizable deep learning models. This study used four publicly available mammography datasets, i.e., DDSM, INbreast, MIAS, and Contrast Limited Adaptive Histogram Equalization (CLAHE)-binary. The CLAHE-binary primary dataset consists of preprocessed mammogram images derived from INbreast, MIAS, and DDSM. This CLAHE-binary dataset was not manually combined in this study and was obtained from Kaggle data (25). To further evaluate the robustness and generalization capability of the proposed frameworks, datasets from Mendeley Data were also utilized (26). These datasets have been previously split into training and validation datasets, with high-quality full-field digital mammograms of biopsy-verified benign and malignant lesions that capture realistic variability acquisition protocols. The DDSM dataset contains 13,128 mammograms that are split into 7,410 and 5,718 training and validation images, respectively. The INbreast dataset includes 6,106 training images and 1,526 validation images, whereas the MIAS dataset comprises 2,954 training images and 862 validation images. Finally, the CLAHE-binary dataset contains 21,283 training images and 5,322 validation images. The samples of mammogram images are shown in Figure 2.

#### 3.1.1 Dataset preparation

All the mammogram images are resized to 224 × 224 pixels, providing a uniform input size for the network. Image resizing ensures dimensional uniformity and compatibility with the input size requirements of architectures. The images are rescaled by a factor of 1/255. Normalization is performed to convert pixel values to the range [0,1], which helps in stable and faster training of the model. Data augmentation operations, including rotation, horizontal flipping, vertical flipping, zooming, shearing, width shifting, and height shifting, are performed to generate additional training samples and reduce overfitting during model training. To prevent data leakage and ensure a fair evaluation, the datasets are divided into 80% training and 20% validation sets, and no samples are used in both sets. The Distribution of datasets is summarized in Table 1.

**Table 1:** Dataset-wise class distribution in training and validation sets.

Datasets	Training images		Validation images		Total images
	Benign	Malignant	Benign	Malignant	
DDSM dataset	3,462	3,948	2,508	3,210	13,128
INbreast dataset	2,106	4,090	504	1,022	7,632
MIAS dataset	1,732	1,222	644	218	3,816
CLAHE-binary dataset	10,312	10,971	2,580	2,742	26,605

### 3.2 Model architecture

To establish a reference performance, two widely used CNN backbones, MobileNetV2 and ResNet50, are first employed as the baseline architectures for binary mammogram classification.

#### 3.2.1 MobileNetV2

MobileNetV2 is a lightweight CNN architecture, specifically designed for mobile and embedded vision applications (12). The ImageNet-pretrained MobileNetV2 is employed as the feature extractor up to its final convolutional feature Maps, which are followed by global average pooling and a compact classification head composed of a 128-unit ReLU layer with dropout. The lower-level convolutional layers are kept frozen, and only the higher-level blocks, together with the classification head, are fine-tuned on the datasets. Several studies have demonstrated that layer freezing during transfer learning can significantly improve training convergence and reduce overfitting by selectively limiting retraining to key layers (27). Despite its compact structure, MobileNetV2 has demonstrated robust performance across various medical imaging tasks, including breast cancer detection.

#### 3.2.2 ResNet50

ResNet50 is a deeper CNN architecture that is initialized with ImageNet-pretrained weights. The architecture consists of a stack of residual blocks with identity shortcuts retained as the feature extractor and final convolutional feature maps are followed by global average pooling and a dense layer with 128 ReLU units with dropout (6). For fine-tuning, the early residual stages are kept frozen, while the upper layers and the classification head are fine-tuned on the CLAHE enhanced mammography dataset to adapt the model to target domains and limit overfitting (17).

### 3.3 Partial fine tuning via layer freezing

Freezing earlier layers in the model is a crucial step of our transfer learning strategy. This technique is motivated by the structure and function of convolutional neural networks, where early layers typically learn the low-level generic features; therefore, these features do not require retraining while adapting to a new model, such as breast cancer classification. These initial layers play an important role in preventing overfitting (19). When models pretrained on large-scale datasets, such as ImageNet, are adapted to specialized medical imaging tasks, retraining these early layers is often unnecessary and may even degrade a model’s performance. By freezing the early layers, the visual representations are preserved, and only the deeper layers are allowed to adapt to task-specific characteristics.

In breast cancer detection, the fine-tuning strategy enables the model to retain reliable low-level feature extraction while learning higher-order patterns associated with malignancy, such as microcalcification clusters, architectural distortion, mass asymmetry, and irregular tissue morphology, which are often subtle and difficult to detect, especially in dense breast tissue. This strategy has benefits when deploying a deep learning system to real-world scenarios, especially as computational resources vary between rural and urban hospitals.

#### 3.3.1 Freeze early layers in the MobileNetV2 architecture

To explore the benefits of transfer learning in the MobileNetV2 architecture for breast cancer classification, some layers are kept frozen. The first 12 layers comprising the initial convolutional layer preserve low-level visual patterns, such as edges, textures, and fine-grain intensity variations, which are important for identifying subtle mammographic characteristics, and the deeper trainable layers adapt to higher-level semantic features, such as tissue asymmetry, masses, and structural abnormalities. This selective transfer learning approach enables the model to retain generalized visual representations while effectively adapting to mammography classification tasks (20, 28). The adopted layer freezing strategy is also beneficial for mammographic feature extraction. The first two bottleneck blocks are frozen during training. The freezing threshold is selected based on the receptive field analysis, with the receptive field remaining localized up to layer 12, which is ideal for detecting the fine-scale structures. From block 13 onward, the receptive field captures broader contextual information, making these layers suitable for learning mass-level patterns and tissue asymmetry. Therefore, the layers from block 13 onward are unfrozen and integrated with SE attention to enable channel-wise feature recalibration in response to CLAHE enhanced-contrast variations. The backbone architecture that follows in this study is adapted from (12) and illustrates the impact of various layer freezing strategies on model convergence and classification accuracy. This resulted in a final MobileNetV2-based model with 2.42 million trainable parameters, achieving a 30.9% reduction compared to the baseline while maintaining high diagnostic sensitivity.

#### 3.3.2 Freezing early layers in the ResNet50 architecture

The first 70 layers of ResNet50 are frozen. The frozen portion of the network consists of early and mid-level feature extractors (18). The initial layers, including the conv1, conv2_x, and conv3_x blocks, are frozen to preserve generic representations, while the deeper stages, i.e., conv4_x and conv5_x, are unfrozen to allow learning of domain-specific mammographic features (29, 30).

The unfrozen conv4_x and conv5_x stages are augmented with SE, which enables joint spatial and channel attention to focus on malignancy, shape irregularities, and contextual asymmetries. The final ResNet50-based model contains 23.85 million trainable parameters, representing a 7% reduction relative to the baseline. The ResNet50-based architectural design adopted in this study follows the structure illustrated in (6), who demonstrated the impact of various layer freezing strategies on model convergence and classification accuracy. This strategy ensures that lower-level representations remain stable while higher-level semantic features are optimized for breast cancer detection.

### 3.4 Attention mechanism

In mammography image analysis, lesions are often subtle and embedded in dense breast tissues, making feature discrimination more difficult for conventional convolutional neural networks. The attention mechanism addresses this challenge by dynamically assigning weights to feature maps, enabling the network to focus on the most relevant features and suppressing the less informative features. Integrating an SE attention mechanism in the network improves channel-wise feature refinement. As a result, the model becomes more sensitive towards the discriminative characteristics of benign and malignant lesions (31).

#### 3.4.1 Integrating the attention mechanism into the MobileNetV2 architecture

**Figure 3:**
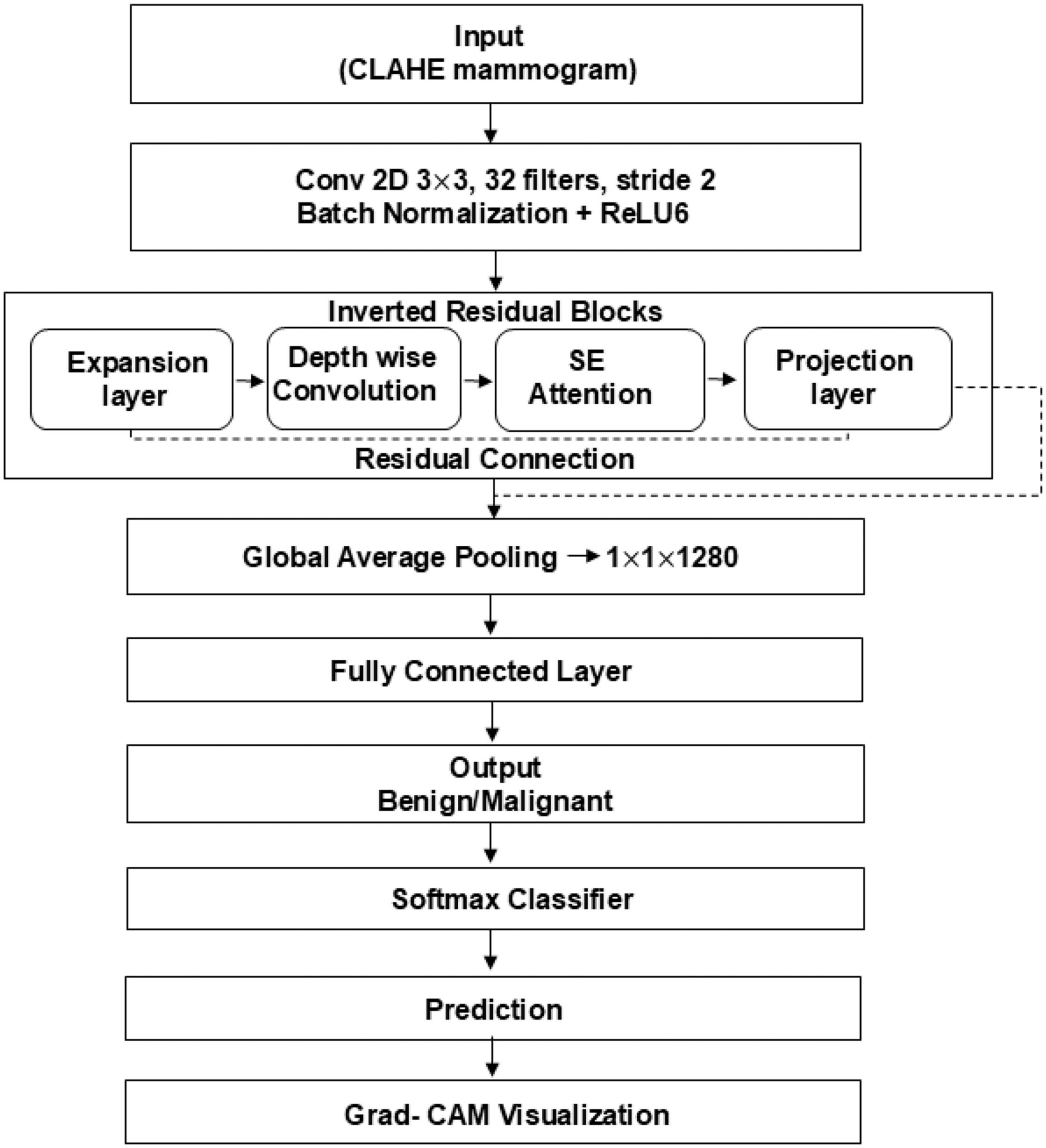
Enhanced MobileNetV2-SE model for breast cancer classification with Grad-CAM visualization for interpretability.

Nirupama and Virupakshappa (32) proposed an enhanced MobileNetV2 framework integrated with SE attention blocks and multi-scale feature aggregation for medical image classification, achieving classification accuracies of 97.62%, 96.45%, 95.13%, and 94.89% across different skin disease datasets, demonstrating the effectiveness of SE attention in improving feature representation and classification performance. In this study, SE attention blocks are integrated into higher stages of the architecture, after bottleneck layer 13. The SE module recalibrates channel-wise feature responses by explicitly learning the relationships between different channels, allowing the network to emphasize informative features, such as high-contrast malignant regions, while suppressing less informative background features more effectively. The extracted features are passed to a regularized binary classification head consisting of global average pooling (GAP), batch normalization, a dense layer with 512 neurons, and ReLU activation, followed by a dropout layer with a rate of 0.5 to reduce overfitting. The final output layer uses a sigmoid activation function to perform a binary classification between benign and malignant cases. The complete MobileNetV2-SE model contains approximately 2.42 million trainable parameters, resulting in a compact model size of approximately 24.62 MB. Figure 3 illustrates the architecture of MobileNetV2-SE model.

#### 3.4.2 Integrating the attention mechanism into the ResNet50 architecture

Jiang et al. (33) proposed a small SE ResNet module for breast cancer histopathological image classification, where the integration of the SE mechanism significantly improved feature representation and achieved classification accuracies ranging from 98.87% to 99.34% for binary classification and 90.66% to 93.81% for multi-class classification tasks. To improve the quality of the channel-wise features in this article, SE is incorporated after the layer freezing stage of ResNet50, where it sequentially applies channel attention to enhance relevant feature representation.

**Figure 4:**
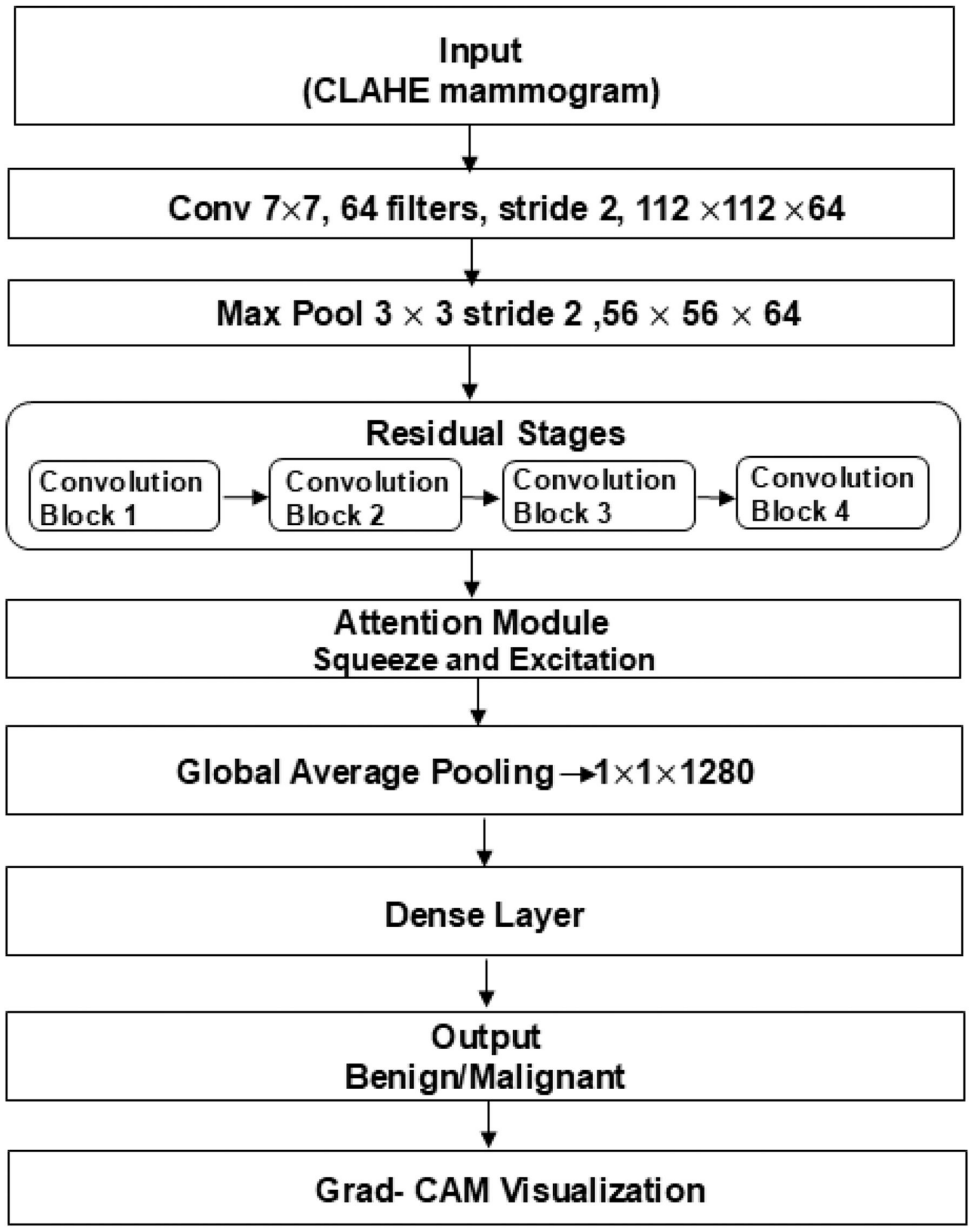
Enhanced ResNet50-SE model for breast cancer classification with Grad-CAM visualization for interpretability.

In this study, the combined ResNet50-SE model uses the regular binary classification head to maintain the uniformity of the model. By keeping the classifier the same, it can directly contribute to the backbone network if there is any performance variation. This dual mechanism is effective for mammograms. The ResNet50-SE architecture consists of 23.85 million parameters with a model size of 224.75 MB and overall architecture of ResNet50-SE is shown in Figure 4.

### 3.5 Explainable AI using Grad-CAM

To improve the interpretability of the models, the Grad-CAM technique is used as an explainable AI approach. Grad-CAM generates a visual heatmap that highlights the important regions of the mammogram images that influence the model’s decision. The Grad-CAM overlay is generated by blending the heatmap with the original image, making it easier to discern which areas the model focuses on when classifying the image as benign or malignant (34). This improves the transparency of the breast cancer detection system. However, these highlighted areas do not necessarily represent precise pathological localization.

**Figure 5:**
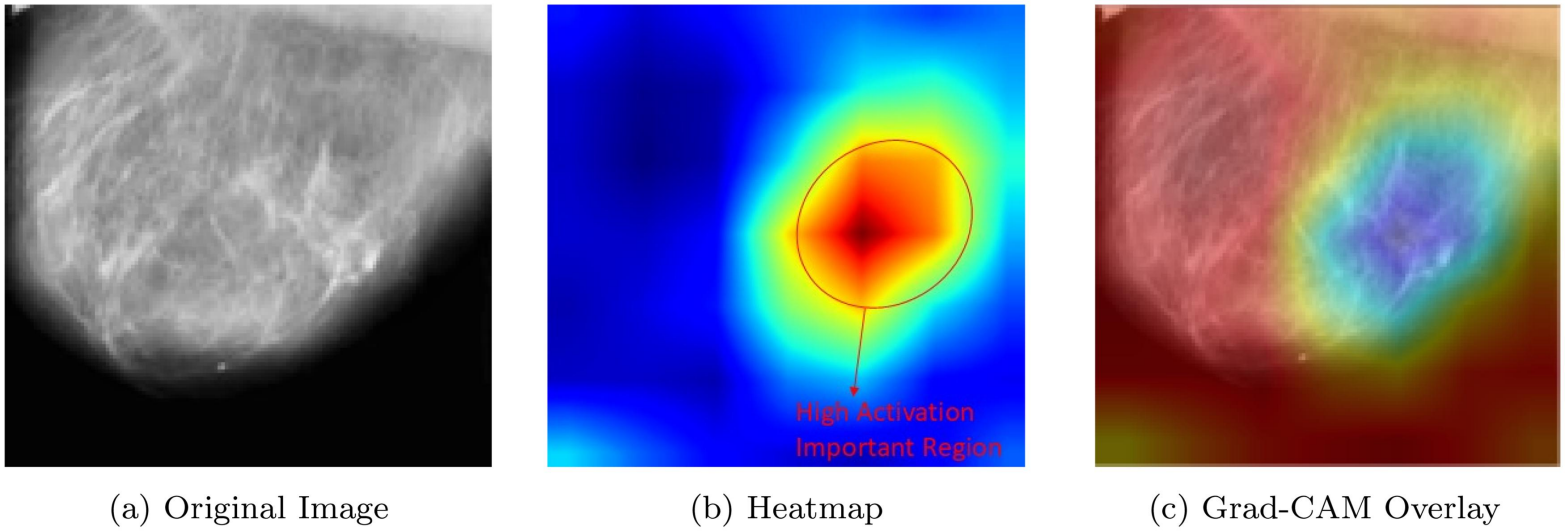
Grad-CAM visualization of the proposed MobileNetV2-SE model, showing **(a)** the original mammogram, **(b)** the generated heatmap, and **(c)** the Grad-CAM overlay highlighting the important regions.

**Figure 6:**
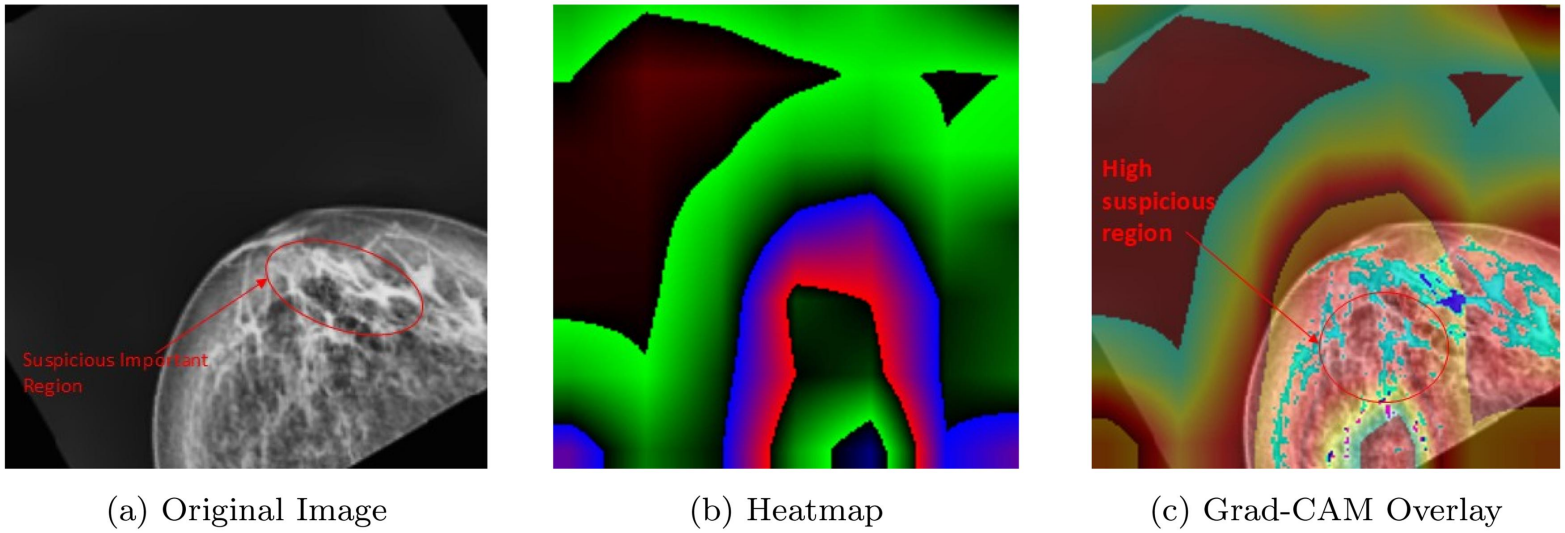
Grad-CAM visualization of the proposed ResNet50-SE model, showing **(a)** the original mammogram, **(b)** the generated heatmap, and **(c)** the Grad-CAM overlay highlighting the important regions.

In this study, Grad-CAM is applied after the training to visualize the models’ predictions. The generated heatmaps that highlight the spatial regions in the network during prediction are shown in Figures 5, 6.

## 4 Results

This section presents the key results obtained using fivefold cross-validation, reported as the mean performance across all folds. To evaluate the stability and robustness of the proposed models, graphical analyses of accuracy, loss, AUC, and the confusion matrix are also included.

## 5 Experimental setup

A laptop was used for the training of the neural networks. The device has 16 GB of random access memory (RAM) and a 512 GB solid-state drive (SSD). The processor is a 13th-generation Intel Core i3. The experiments were accelerated using the NVIDIA Tesla T4 GPU support available in Google Colab. Early stopping was applied to monitor the validation AUC during the initial training and fine-tuning. The programming was conducted using the Python language along with TensorFlow and Keras libraries for deep learning model development and training. Table 2 shows the initial parameters for both models during training.

**Table 2:** Initial parameters for MobileNetV2-SE and ResNet50-SE.

Parameter	Value/type
Input size	224×224×3
Optimizer	Adam optimizer
Learning rate	0.001
Fine-tuning learning rate	0.00001
Batch size	32
Output activation function	Sigmoid

The statistical analysis of the models was performed using standard metrics: precision, recall, F1 score, accuracy, and loss. The models were trained for 20 epochs as the validation performance reached saturation within this range. Increasing the number of epochs resulted in negligible improvement, while increasing computational cost and the risk of overfitting.

### 5.1 Performance parameters

The performance parameters are the calculated figures that help in evaluating an architecture’s performance and how well it converges. The following performance parameters were used to assess the performance of the models.

- Confusion matrix: The confusion matrix provides a tabular summary of the predicted and actual class labels. It can be utilized to compute numerous evaluation parameters, such as accuracy, precision, and recall. The rows represent the predicted classes, while the columns denote the actual classes. The diagonal elements reflect cases that have been correctly classified, whereas the off-diagonal elements indicate cases that have been incorrectly classified. The confusion matrix itself can be utilized to compute the recall and precision values for each label.
  The confusion matrix terminology can be explained as follows:
  - True positive: Prediction = 1 and actual output = 1.
  - True negative: Prediction = 0 and actual output = 0.
  - False positive: Prediction = 1 and actual output = 0.
  - False negative: Prediction = 0 and actual output = 1.
- Accuracy: This is the most used evaluation parameter for classification models. It measures the percentage of correctly classified cases among total cases. It can be determined using Equations (1–3).:
  $$\text{Accuracy}=\frac{\text{TP}+\mathrm{TN}}{\mathrm{TP}+\mathrm{TN}+\mathrm{FP}+\mathrm{FN}}$$ (1)
  $${\mathrm{Accuracy}}_{\mathrm{kfold}}=\frac{1}{K}\sum_{i=1}^{K}{\mathrm{Accuracy}}_{i}$$ (2)
  $$\sigma_{\mathrm{Accuracy}}=\sqrt{\frac{1}{K}\sum_{i=1}^{K}({\mathrm{Accuracy}}_{i}-\mu {)}^{2}}$$ (3)
- Precision: Precision is the percentage of correctly classified cases that are positive relative to the total number of cases that were classified as positive. It can be calculated as Equation (4–6):
  $$\mathrm{Precision}=\frac{\text{TP}}{\text{TP}+\mathrm{FP}}$$ (4)
  $${\mathrm{Precision}}_{\mathrm{kfold}}=\frac{1}{K}\sum_{i=1}^{K}{\mathrm{Precision}}_{i}$$ (5)
  $$\sigma_{\mathrm{Precision}}=\sqrt{\frac{1}{K}\sum_{i=1}^{K}({\mathrm{Precision}}_{i}-\mu {)}^{2}}$$ (6)
- Recall: Recall is the percentage of correctly classified positive cases out of all the positive cases. It can be calculated as Equation (10–12):
  $$\mathrm{Recall}=\frac{\text{TP}}{\text{TP}+\mathrm{FN}}$$ (7)
  $${\mathrm{Recall}}_{\mathrm{kfold}}=\frac{1}{K}\sum_{i=1}^{K}{\mathrm{Recall}}_{i}$$ (8)
  $$\sigma_{\mathrm{Recall}}=\sqrt{\frac{1}{K}\sum_{i=1}^{K}({\mathrm{Recall}}_{i}-\mu {)}^{2}}$$ (9)
- F1 score: The F1 score is the average of the precision and recall. It offers a balance between precision and recall and is frequently employed when classes are imbalanced. It can be calculated as follows:
  $$\text{F1 score}=2*\frac{\text{\textbackslash{},precision}*\text{recall}}{\text{\textbackslash{},precision}+\text{recall}}$$ (10)
  $$F1_{\mathrm{kfold}}=\frac{1}{K}\sum_{i=1}^{K}F1_{i}$$ (11)
  $$\sigma_{F1}=\sqrt{\frac{1}{K}\sum_{i=1}^{K}(F1_{i}-\mu {)}^{2}}$$ (12)
- AUC: The AUC is the area under the ROC curve and evaluates the overall classification capability of the model. It is calculated as Equation (13–15):
  $$\mathrm{AUC}=\int_{0}^{1}\mathrm{TPR}({\mathrm{FPR}}^{-1}(x))\;dx$$ (13)
  $${\mathrm{AUC}}_{\mathrm{kfold}}=\frac{1}{K}\sum_{i=1}^{K}{\mathrm{AUC}}_{i}$$ (14)
  $$\sigma_{\mathrm{AUC}}=\sqrt{\frac{1}{K}\sum_{i=1}^{K}({\mathrm{AUC}}_{i}-\mu {)}^{2}}$$ (15)
- Training loss: This is the error rate on the training dataset. It is the measure of the difference between the predicted output and the actual output on the training set.
- Validation loss: The error rate on the validation dataset after training.

### 5.2 Quantitative results

**Figure 7:**
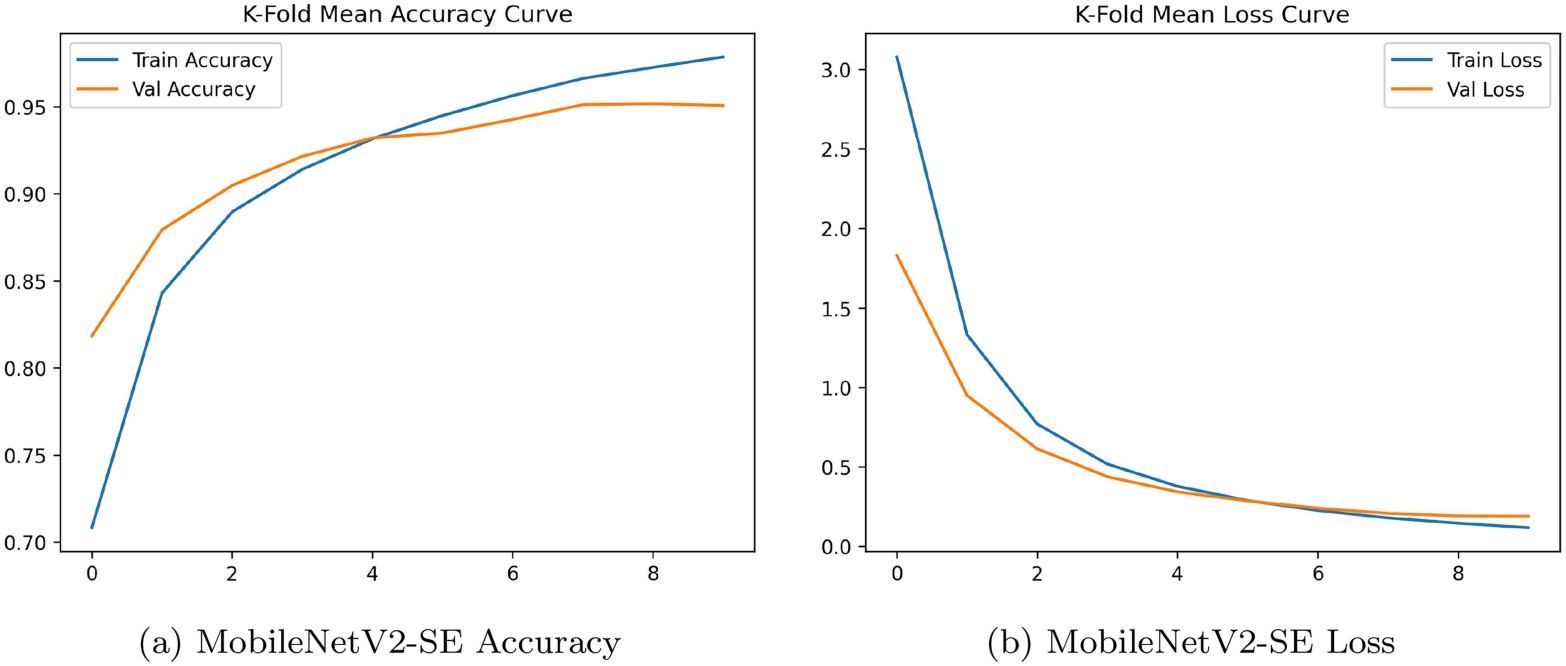
Mean training and validation curves obtained using 5-fold cross-validation for MobileNetV2-SE. (**a**) Accuracy curve showing training and validation accuracy. (**b**) Loss curve showing training and validation loss.

**Figure 8:**
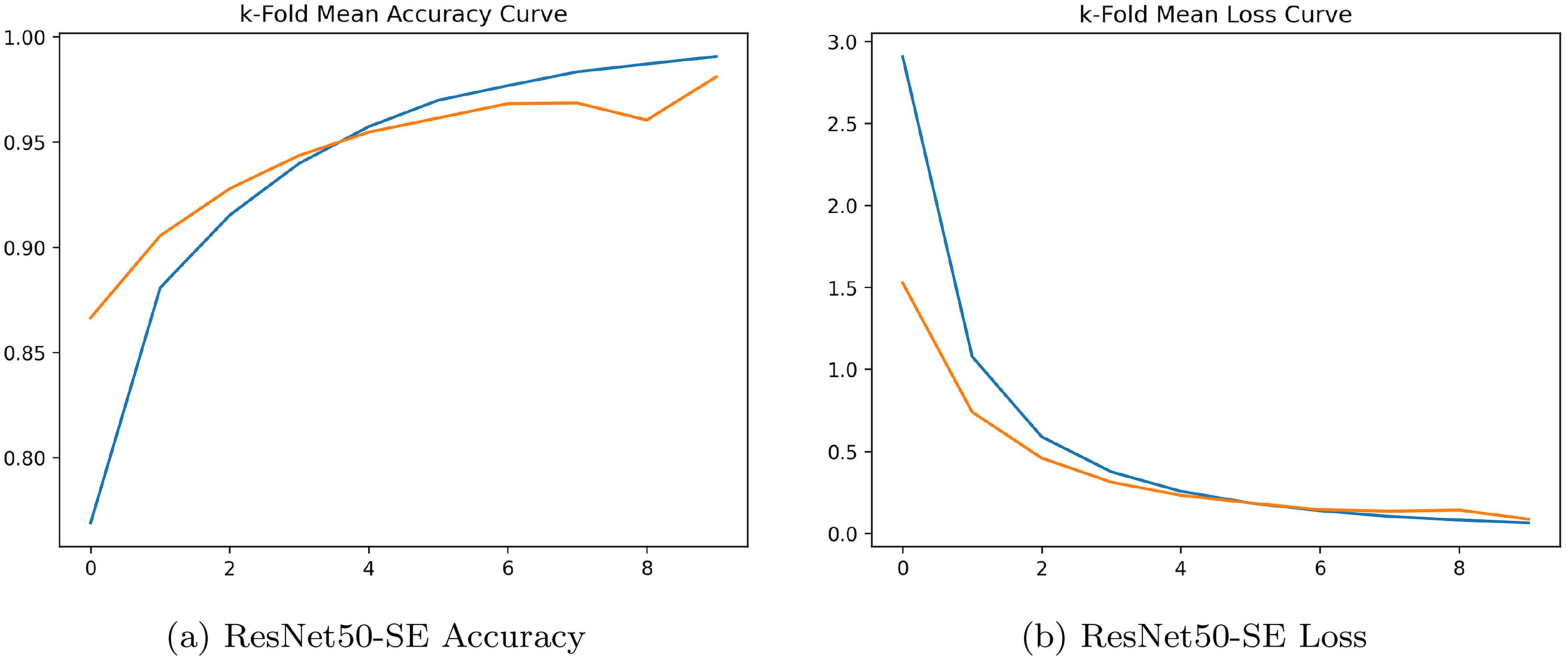
Mean accuracy and loss curves obtained using 5-fold cross-validation for ResNet50-SE model. (**a**) Accuracy curve showing training and validation accuracy. (**b**) Loss curve showing training and validation loss.

The training and validation accuracy and loss curves obtained using fivefold cross-validation, computed as the mean performance across all folds, are illustrated in Figures 7, 8, respectively. Both MobileNetV2-SE and ResNet50-SE achieved higher validation accuracy, which indicates that the learning capacity in the deeper architecture was improved. Both models showed stable convergence without overfitting. The loss curves decreased smoothly and confirm effective optimization for both backbones.

**Figure 9:**
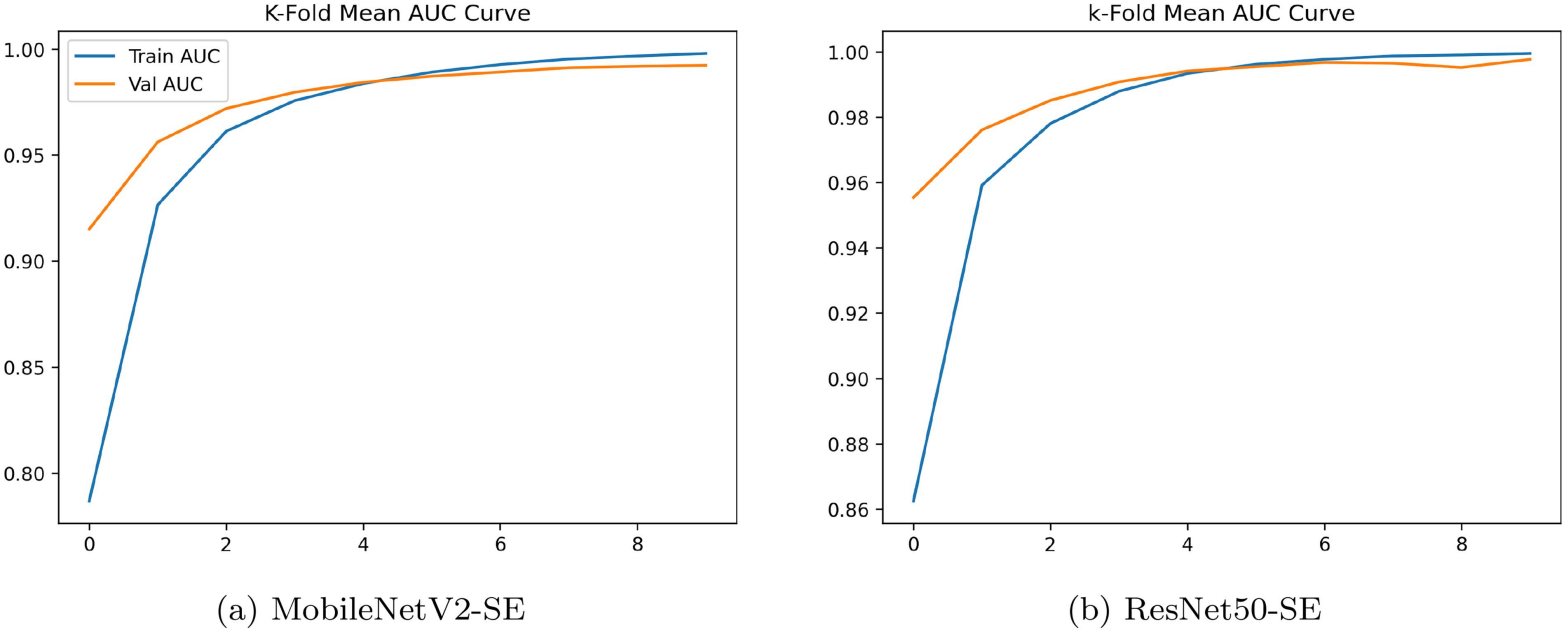
AUC curves of (**a**) MobileNetV2-SE and (**b**) ResNet50-SE.

Using fivefold cross-validation, the MobileNetV2-SE model achieved an AUC of 0.9944 on the CLAHE-binary dataset, despite its lightweight design, demonstrating string discriminative ability. The ResNet50-SE yielded an AUC of 0.9984 on the CLAHE-binary dataset, as shown in Figure 9. The results clearly show that both models maintained high true positive rates compared to false positives, making them more robust and passing the clinically applicable threshold.

**Figure 10:**
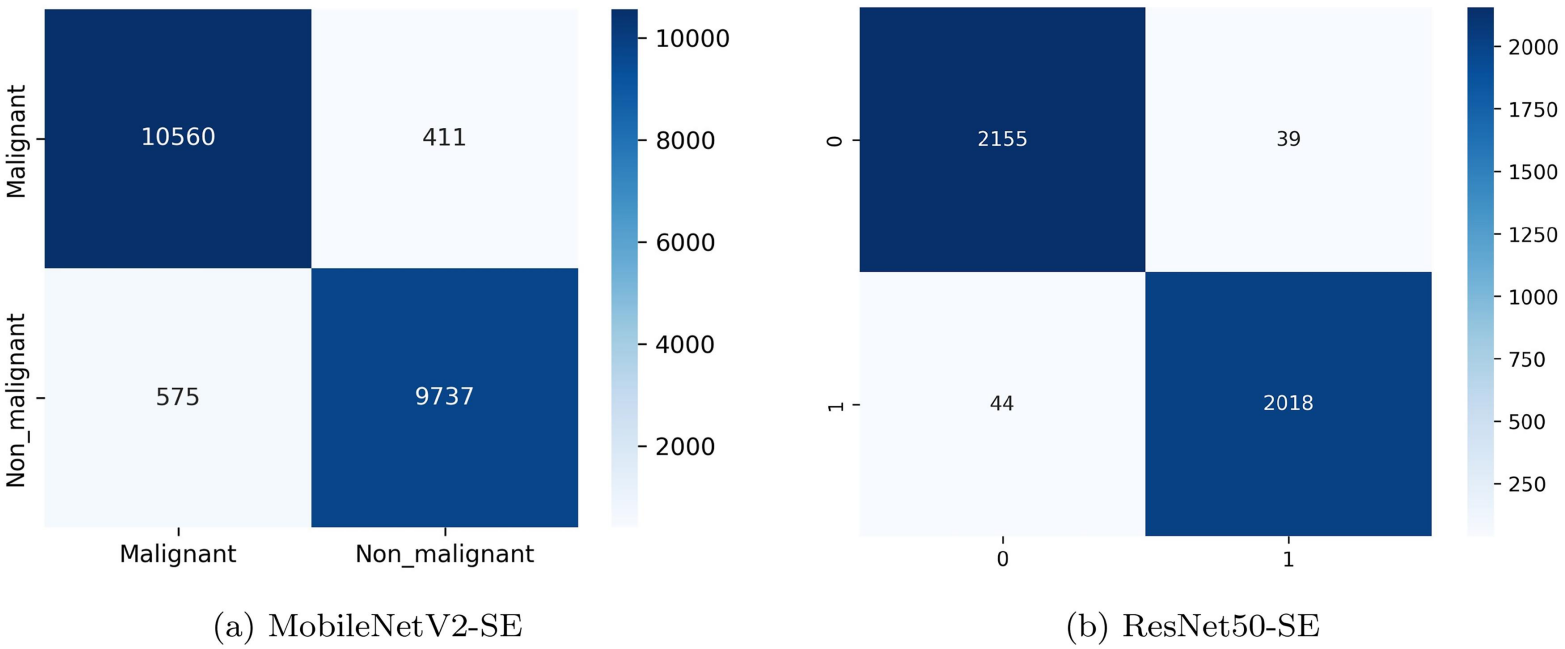
Confusion matrices of (**a**) MobileNetV2-SE and (**b**) ResNet50-SE models.

In addition to the overall accuracy, loss, and AUC analyses, a confusion matrix and evaluation metrics were used to further assess the performance of the proposed models using fivefold cross-validation, as shown in Figure 10. The confusion matrix provides insight into the distribution of false positive and false negative classifications of benign and malignant samples. The precision, recall, and F1 score evaluate the balance between false positive and false negative predictions, which is particularly important in medical image analysis.

The class-wise metrics validate the performance of the proposed models. The high precision and recall values indicate low false positive and false negative rates, while the balanced F1 score confirms consistent classification performance across both classes. To ensure a more reliable and unbiased evaluation of the proposed models, fivefold cross-validation was employed. This evaluation strategy helps reduce dependency on a single train test split and provides a more stable estimate of the models’ performance. Therefore, the reported results in Table 3 include the mean performance along with standard deviation, which reflects the consistency of the models across different datasets.

**Table 3:** Average fivefold cross-validation performance of the proposed models across different datasets.

Dataset	Accuracy	Precision	Recall	F1 score	AUC
MobileNetV2-SE					
DDSM	0.9914 ± 0.0028	0.9899± 0.0001	0.9939± 0.0001	0.9919 ± 0.0001	0.9995 ± 0.0003
INBreast	0.9353 ± 0.0145	0.9416 ± 0.0265	0.9644 ± 0.0188	0.9524 ± 0.0101	0.9841 ± 0.0049
MIAS	0.9235 ± 0.0140	0.8983 ± 0.0314	0.9021 ± 0.0545	0.8984 ± 0.0212	0.9776 ± 0.0050
CLAHE combined	0.9633 ± 0.0055	0.9665 ± 0.0028	0.9574 ± 0.0118	0.9619 ± 0.0059	0.9944 ± 0.0010
ResNet50-SE					
DDSM	1.0000 ± 0.0000	1.0000 ± 0.0000	1.0000 ± 0.0000	1.0000 ± 0.0000	1.0000 ± 0.0000
INBreast	0.8889 ± 0.0156	0.8808 ± 0.1267	0.4123 ± 0.3974	0.4173 ± 0.3203	0.7634 ± 0.035
MIAS	0.7160 ± 0.0139	0.6590 ± 0.0464	0.6775 ± 0.0910	0.6610 ± 0.0236	0.8016 ± 0.0245
CLAHE combined	0.9798 ± 0.0022	0.9799 ± 0.0080	0.9786 ± 0.0091	0.9792 ± 0.0023	0.9984 ± 0.0004

### 5.3 Explainability analysis using Grad-CAM

Grad-CAM visualizations are shown in Figures 5, 6, with different colors representing the level of importance assigned by the model. The red and yellow regions indicate highly important areas, showing where the model strongly focuses for classification, while green regions are moderately important, and blue regions have a low contribution to the prediction. This visualization helps us interpret how the model identifies discriminative regions in mammograms.

## 6 Discussion

In this study, we present a comparative analysis of two deep learning-based models for breast cancer detection using mammogram datasets. Table 4 presents the comparative analysis of the proposed frameworks, namely, MobileNetV2-SE and ResNet50-SE, with recent deep learning-based approaches to breast cancer classification. MobileNet2-SE achieved a more stable performance than ResNet50-SE across limited data datasets. The compact feature representation of MobileNetV2-SE may help reduce overfitting and improve generalization performance, increasing its ability to accurately classify benign and malignant lesions. ResNet50-SE achieved good results due to its residual connections. However, some performance variability was observed across the MIAS and Inbreast datasets, possibly due to differences in dataset characteristics, image quality, and limited sample availability.

**Table 4:** Performance comparison of the proposed frameworks with existing breast cancer classification approaches.

Study	Model	Dataset	Modality	Accuracy (%)	Precision	Recall
Shim et al. (5)	InceptionResNetV2	Mammogram	Mammography	97.94	0.9806	0.9939
Alshamrani et al. (6)	ResNet50	BI-RADS (balanced)	Mammography	95.0	0.98	0.96
		BI-RADS (unbalanced)	Mammography	90.0	0.96	0.74
Taifi et al. (12)	MobileNetV2	DDSM	Mammography	99.4	0.99	0.99
		INbreast	Mammography	99.0	0.98	0.99
		MIAS	Mammography	99.0	0.98	0.97
Taifi et al. (12)	DenseNet121	DDSM	Mammography	99.4	0.99	0.99
		INbreast	Mammography	98.0	0.98	0.99
		MIAS	Mammography	98.0	0.98	0.97
Ahmed and Nandi (40)	MoEffNet	INbreast/CBIS-DDSM	Mammography	99.8	0.998	0.997
Proposed	MobileNetV2-SE	DDSM	Mammography	**99.95**	**1.00**	**1.00**
		INbreast	Mammography	**95.28**	**0.97**	**0.96**
		MIAS	Mammography	**67.63**	**0.75**	**0.82**
		CLAHE combined	Mammography	**99.36**	**0.99**	**0.99**
Proposed	ResNet50-SE	DDSM	Mammography	**96.47**	**0.94**	**1.00**
		INbreast	Mammography	**74.25**	**0.78**	**0.86**
		MIAS	Mammography	**62.53**	**0.72**	**0.81**
		CLAHE combined	Mammography	**88.67**	**0.85**	**0.94**

Bold values indicate the results of the MobileNetV2-SE and ResNet50-SE.

The existing literature shows that the majority of approaches rely on standard convolutional or transfer learning architectures. Taifi et al. (12) achieved strong performance using MobileNetV2 in multiple mammography datasets, including DDSM, INbreast, and MIAS. The higher accuracy indicates the effectiveness of transfer learning for feature extraction in medical imaging. However, the model does not explicitly incorporate advanced feature refinement or adaptive attention mechanisms, which may limit its ability to focus on subtle lesion-specific regions. Rakha et al. (13) proposed a MobileNetV2 CBAM-based architecture for ultrasound image classification. Although the inclusion of CBAM introduces channel and spatial attention, the overall improvement remains constrained by the lightweight backbone and limited hierarchical feature depth, which may restrict complex feature representation in challenging cases, whereas Alshamrani et al. (6) utilized ResNet50 architectures for Breast Imaging Reporting and Data System (BI-RADS) classification in both balanced and unbalanced datasets.

While ResNet50 provides a strong deep feature extraction capability, the noticeable drop in performance in unbalanced data settings highlights sensitivity to dataset distribution and class imbalances, indicating limited robustness for real-world clinical scenarios. Zeng et al. (35) reported very high performance using a modified ResNet-based architecture on the BreaKHis histopathology dataset. Although the method shows good results for histopathological image classification, its performance is largely domain-specific and may not directly generalize to other modalities, such as mammography or ultrasound imaging, due to differences in feature characteristics.

In contrast, the proposed frameworks capture hierarchical spatial representations from medical images using weights learned from large-scale datasets. This transfer learning strategy improves convergence stability and enables the extraction of highly discriminative low-level and high-level features. Both models were trained with fine-tuning of pretrained ImageNet weights, allowing for adaptation to mammograms. The attention block plays a critical role in improving feature quality by adaptively recalibrating channel-wise feature responses. The global pooling and fully connected classification layers transform the refined feature maps into compact feature vectors and perform final decision mapping. These layers improve class separability and reduce overfitting by minimizing spatial redundancy in feature representations.

As a result, the proposed approaches outperform the existing methods in the literature in terms of accuracy, precision, recall, and F1 score. Furthermore, the SE attention mechanism enhances feature discrimination by emphasizing diagnostically relevant regions (36–38).

Although some previous studies have reported higher accuracies in smaller datasets by employing extensive data augmentation (39), synthetic oversampling and cross-validation strategies can artificially boost performance without a corresponding improvement in real-world generalization. In comparison, this study evaluates the proposed models under more constrained and realistic conditions, prioritizes dataset-level separation, and avoids artificial data inflation, as shown in Table 4.

In addition, variations in image quality and lesion appearance across datasets, certain mammographic patterns, misclassification, low-contrast lesions, irregular tissue structures, and overlapping anatomical regions where abnormal patterns are less visually distinguishable may influence prediction consistency. These observations highlight the complexity of mammographic analysis and indicate the need for further improvements in model generalization and feature discrimination. This study highlights the need for larger annotated datasets or advanced data-centric strategies to fully leverage deep learning models in breast cancer detection.

### 6.1 Ablation study on SE attention mechanism

The comparative analysis presented in Table 5 demonstrates that the integration of the SE attention mechanism influenced the baseline models’ performance differently in the evaluated datasets. In several cases, SE enhanced the models’ performance, achieving better accuracy and improving feature extraction. However, for some datasets, the improvement was limited, which may be due to variations in the dataset characteristics, image quality, and class distribution.

**Table 5:** Comparative analysis of the baseline and SE attention-enhanced models across multiple datasets for breast cancer classification.

Model	Dataset	Accuracy (%)	Loss (%)	AUC
Baseline models				
MobileNetV2	DDSM	99.91	0.66	1.000
MobileNetV2	INBreast	89.91	26.45	0.984
MobileNetV2	MIAS	62.99	10.64	0.517
MobileNetV2	CLAHE-Binary	98.58	0.90	0.998
ResNet50	DDSM	99.13	0.25	0.997
ResNet50	INBreast	70.12	52.80	0.801
ResNet50	MIAS	71.11	10.98	0.335
ResNet50	CLAHE-Binary	89.01	25.65	0.964
Attention integrated models				
MobileNetV2-SE	DDSM	99.95	0.21	0.999
MobileNetV2-SE	INBreast	95.28	12.45	0.984
MobileNetV2-SE	MIAS	67.63	13.46	0.977
MobileNetV2-SE	CLAHE-Binary	99.36	0.13	0.994
ResNet50-SE	DDSM	96.47	0.10	1.000
ResNet50-SE	INBreast	74.25	24.67	0.763
ResNet50-SE	MIAS	62.53	10.00	0.801
ResNet50-SE	CLAHE-Binary	88.67	0.21	0.998

## 7 Conclusions

In this study, the performance of two transfer learning-based models, namely, MobileNetV2 and ResNet50, with SE attention mechanisms were evaluated for breast cancer classification. The robustness and generalizability of the proposed models were further validated by a comparative analysis performed on different mammography datasets. Furthermore, the incorporation of SE attention mechanisms helped the networks to pay increased attention to the most important regions in the mammographic images, resulting in better learning features and classification performance. In addition, this study shows that lightweight models can deliver consistent and stable performance with a limited dataset and thus have the potential to be practical in real-world environments. MobileNetV2-SE showed better performance with improved accuracy, precision, and recall values on the DDSM, INbreast, MIAS, and CLAHE combined dataset. Moreover, the obtained results for ResNet50 on the smaller dataset, MIAS, were comparable with existing methods.

Future research should focus on increasing the size of the dataset and incorporating balancing strategies. Further improvements may also be achieved by investigating alternative attention mechanisms and adaptive fine-tuning strategies that dynamically select trainable layers instead of relying on a fixed freezing ratio. Extending the evaluation to multi-class classification and cross-dataset validation would provide deeper insights into the clinical reliability and real-world applicability of the proposed frameworks.

## Data availability statement

The original contributions presented in the study are included in the article/Supplementary Material, further inquiries can be directed to the corresponding author.

## Ethics statement

Ethical approval was not required for this study involving humans in accordance with the local legislation and institutional requirements. Written informed consent to participate in this study was not required from the participants or the participants’ legal guardians/next of kin in accordance with the national legislation and the institutional requirements.
